# Evaluation of Diagnostic Performance of Three Indirect Enzyme-Linked Immunosorbent Assays for the Detection of IgG Antibodies to Ebola Virus in Human Sera

**DOI:** 10.3390/v11080678

**Published:** 2019-07-24

**Authors:** Janusz T. Paweska, Naazneen Moolla, Nadia Storm, Veerle Msimang, Ousman Conteh, Jacqueline Weyer, Petrus Jansen van Vuren

**Affiliations:** 1 Centre for Emerging Zoonotic and Parasitic Diseases, National Institute for Communicable Diseases of the National Health Laboratory Service, Sandringham, Johannesburg 2131, South Africa; 2 Faculty of Health Sciences, University of the Witwatersrand, Johannesburg 2050, South Africa; 3 Ministry of Health and Sanitation, Freetown 47235, Sierra Leone

**Keywords:** Ebola virus, enzyme-linked immunosorbent assay, whole antigen, nucleocapsid, glycoprotein, IgG antibody, human serum, diagnostic performance

## Abstract

Filovirus serological diagnosis and epidemiological investigations are hampered due to the unavailability of validated immunoassays. Diagnostic performance of three indirect enzyme-linked immunosorbent assays (I-ELISA) was evaluated for the detection of IgG antibody to Ebola virus (EBOV) in human sera. One I-ELISA was based on a whole EBOV antigen (WAg) and two utilized recombinant nucleocapsid (NP) and glycoproteins (GP), respectively. Validation data sets derived from individual sera collected in South Africa (SA), representing an EBOV non-endemic country, and from sera collected during an Ebola disease (EBOD) outbreak in Sierra Leone (SL), were categorized according to the compounded results of the three I-ELISAs and real time reverse-transcription polymerase chain reaction (RT-PCR). At the cut-off values selected at 95% accuracy level by the two-graph receiver operating characteristic analysis, specificity in the SA EBOV negative serum panel (*n* = 273) ranged from 98.17% (GP ELISA) to 99.27% (WAg ELISA). Diagnostic specificity in the SL EBOV negative panel (*n* = 676) was 100% by the three ELISAs. The diagnostic sensitivity in 423 RT-PCR confirmed EBOD patients was dependent on the time when the serum was collected after onset of disease. It significantly increased 2 weeks post-onset, reaching 100% sensitivity by WAg and NP and 98.1% by GP I-ELISA.

## 1. Introduction

High-mortality and occurrence of Ebola disease (EBOD) outbreaks almost each year in the last three decades [1,2] are of great public health concern. The unprecedented and first epidemics of EBOD in West Africa from 2013 to 2016 [3,4,5,6] and the large-scale re-emergence of EBOD in the Democratic Republic of the Congo in 2018 and 2019 [2,7] exemplify the devastating health, humanitarian, and socio-economic impacts and challenges in containing EBOD outbreaks in resource-poor and politically conflicted settings [5,6,7,8,9,10,11,12,13,14].

The increasing incidence, severity and size of EBOD outbreaks highlight the importance of developing and standardizing diagnostic tools for EBOD rapid diagnosis and post-epidemic surveillance. The most devastating EBOD outbreak to date, the West African outbreak, prompted the development of diagnostic assays that can address limited laboratory infrastructure, resources and personnel in affected areas and fulfill requirements for point-of-care (POC) rapid diagnostic tests (RDTs), including real-time reverse-transcription polymerase chain reaction (RT-PCR) based on Cepheid GeneXpert technology [15,16] and antigen detection lateral flow immunoassays [17,18]. While Ebola virus (EBOV) POC and RDTs molecular and virological methods have been successfully developed and evaluated, serological diagnosis, disease serosurveillance and control programs and monitoring of the immune responses in vaccines remain a challenging subject due to unavailability of validated serological assays. Collection of diagnostic specimens after viral clearance and inappropriate transportation methods and storage conditions may negatively impact molecular assays [19,20] making serology testing an important tool in the public health response to outbreaks occurring in remote locations where limited resources are available. Serological tests such as enzyme-linked immunosorbent assays (ELISA) are used for the detection of antibody responses to antigens associated with filovirus infection or vaccination. Establishment of such assays is dependent on the production of high quality immunoreagents [21]. Recombinant antigen technology offers the advantage of development and production under biosafety level two conditions.

Recombinant filoviral nucleoprotein (NP) is an ideal target for ELISAs due to their high immunogenicity and abundance in infected cells [22]. The NPs of filoviruses are highly conserved [23], and ELISAs based on recombinant EBOV NP antigens have been reported to cross-react with sera from humans and animals infected with different filovirus species [24,25,26]. Recombinant NPs are therefore useful for genus-specific screening ELISAs where the specific filovirus species with which a patient is infected is unknown [24,26]. The glycoprotein (GP) is another ideal antigen for filovirus ELISAs as it contains epitopes known to be targets for filovirus-neutralizing antibody responses [27]. Contrary to the NP, the GP is highly diverse [23] and is useful for ELISAs where the main purpose is to detect species-specific filoviral antibodies.

Recombinant protein-based ELISAs for filoviruses were first developed by Prehaud and colleagues in 1998 [28]. The assays made use of either recombinant NP antigens expressed in an *Escherichia coli* (*E. coli*) system, or recombinant GP antigens expressed in a baculovirus-*Spodoptera frugiperda* (Sf9) cell expression system. The antigens reacted positively with sera from previously EBOV-infected human patients [28]. Several other groups have since used and adapted these protein expression techniques for the development of serological assays for EBOV and have reported high sensitivity and specificity for the detection of anti-EBOV IgG [24,29,30,31]. However, recombinant antigens expressed in a bacterial expression system are often problematic due to a lack of molecular folding and post-translational modifications such as glycosylation [32]. Recombinant antigens expressed in baculovirus-insect cell systems prevail over some of the problems presented by those expressed in bacterial expression systems, but the antigenic properties of these antigens may be considerably affected by the differing glycosylation pathways between insect and mammalian cells [33]. To overcome the above-mentioned problems, Nakayama and colleagues [26] developed the first filovirus ELISAs based on recombinant Histidine-tagged GP expressed in human embryonic kidney 293T cells using the mammalian expression vector pCAGGS-MCS [26]. Vu and colleagues developed, optimized, and evaluated a similar ELISA for the quantification of antibodies to EBOV in vaccinated non-human primates, but human samples were unavailable for testing [31]. A field-validated ELISA based on commercially available recombinant EBOV GP has been reported for the detection of immunoglobulin G (IgG) in oral fluid [34]. However, due to the lower sensitivity of oral fluid-based assays compared to serum-based assays, there is a risk of misdiagnosing patients with a low oral concentration of anti-EBOV IgG.

Although a number of new generation filovirus serological assays have been developed in recent years, none have been adequately validated to date for the detection of IgG in sera from EBOV-infected humans. This is largely due to the difficulty in obtaining panels of clinical material sufficient in size to allow for statistically sound evaluation. Nevertheless, the validation of serological assays and determination of their basic diagnostic performance parameters are essential for reporting diagnostic results, comparing patient results between different diagnostic laboratories, evaluating ELISA efficacies, determining seroprevalence rates, infection risk population studies, and assessing occurrence of asymptomatic infections [35].

The purpose of this study was to evaluate and compare the diagnostic performance of EBOV IgG-indirect ELISAs based on antigens produced by classical virological and recombinant protein expression methods using human serum panels from EBOV non-infected and EBOV infected humans.

## 2. Materials and Methods

### 2.1. Specimens and Ethics Clearance

A total of 1372 individual banked sera collected in South Africa (SA) and Sierra Leone (SL), were used. SA sera (*n* = 273) were originally submitted for various routine diagnostic testing to the Centre for Emerging Zoonotic and Parasitic Diseases of the National Institute for Communicable Diseases (NICD), Johannesburg. These sera represented specimens collected from 273 individuals in EBOV non-endemic country and are regarded as IgG EBOV negative reference serum panel. Ethics clearance for using SA human banked sera in the development and validation of diagnostic assays was obtained from Human Ethics Committee, University of the Witwatersrand, Johannesburg, SA, Clearance Certification No. M1809219, 23 February 2018.

SL blood specimens were originally submitted for EBOV RT-PCR testing to the SA modular high-biosafety field Ebola diagnostic laboratory (FEDL) established in August 2014 near Freetown, in international response to the rapidly increasing number of EBOD cases in SL [36]. Selected aliquots of processed sera were shipped on dry ice from FEDL to the NICD’s biosafety level 4 (BSL-4) facility in Johannesburg, SA by the end of March 2015 for long-term storage and post-outbreak investigations. Export permits were obtained from the Pharmacy Board of SL (Nos.: PBSL/061/02 2015, PBSL/061/03 2015). Ethics clearance for using SL human sera in the development and validation of EBOV diagnostic assays was obtained from the Government of Sierra Leone, Office of the Sierra Leone Ethics and Scientific Review Committee, Directorate of Training and Research, Ministry of Health and Sanitation (application version of 12 October 2015, clearance issued on 02 November 2015 and 16 October 2018). The ethics identification codes were not provided on ethics clearances issued by the Office of the Sierra Leone Ethics and Scientific Review Committee.

In total, 1099 sera obtained from SL patients suspected of having EBOD between August 2014 and March 2015 [36] were used. Of those sera 423 were from EBOV RT-PCR confirmed cases for whom date of disease onset was recorded on EBOD case submission form. Ribonucleic acid from blood was extracted using the QIAamp viral RNA kit (Qiagen, Hilden, Germany) according to the manufacturer’s instructions. RT-PCR was performed using the QIAGEN One-Step RT-PCR kit (Qiagen, Hilden, Germany) as per the manufacturer’s instructions using previously described primers and probes targeting the EBOV L gene [37]. Specimens with *Ct* values below 40 were considered positive for EBOV RNA [36] and thus confirming EBOV infectious status of a patient. Serum specimens from the SL EBOV RT-PCR positive patients were regarded as SL reference positive serum panel.

The remaining specimens were from 676 EBOV RT-PCR negative individuals whose sera tested negative for anti-EBOV IgG by all IgG ELISAs evaluated in this study using cut-offs derived from SA IgG EBOV negative reference serum panel. This serum panel was regarded as SL reference negative serum panel.

### 2.2. ELISA Serum Controls and Internal Quality Control (IQC)

The source of positive control serum (C++) was the 1996 imported EBOD case from Gabon to SA [38,39]. The case was a Gabonese physician who had been brought from Libreville, Gabon on 27 October and admitted to a private hospital in Johannesburg where a nurse died after being infected through exposure to his blood.

Negative control serum (reference no. CEZPD SVPL 502/2017) was obtained from South African Blood Service. It tested negative for anti-EBOV IgG and IgM antibodies by in-house ELISA using procedures published by Ksiazek et al. [40].

Internal quality control (IQC) data were generated as described previously [41]. Upper and lower control IQC limits together with coefficients of variations ≤10% for replicates of positive internal control serum and test sera were applied as an assay acceptance criteria.

### 2.3. Enzyme-Linked Immunosorbent Assays

Diagnostic performance of three indirect ELISAs (I-ELISA) for the detection of anti-EBOV IgG antibody in human sera was evaluated. These ELISAs were based on antigens prepared from infected cell lysate, or on recombinant antigens produced in a bacterial or mammalian expression system.

#### 2.3.1. Production of Ebola Virus Whole Antigen

The EBOV whole antigen (WAg) was produced as previously described with some modifications [40]. Vero C1008 cells (ATCC, Manassas, VA, USA) were infected with the SPU 220/96 isolate of EBOV (4th passage in Vero cells) isolated from the serum of a nurse who contracted a fatal infection from a Gabonese physician admitted to a private hospital in South Africa in 1996 [39]. After incubation at 37 °C when cytopathic effect was observed, cells and supernatant were collected and snap freeze-thawed at −70 °C and 37 °C. Lysed cells and supernatant were separated by centrifugation (10,000× *g*, 4 °C, 15 min). The collected supernatant was gamma irradiated with 30,000 Gy to inactivate the virus. Saturated ammonium sulphate solution (100%) (Sigma Aldrich, Merck, Kenilworth, NJ, USA) was slowly added with constant stirring to the irradiated supernatant to a final concentration of 35%, and the mixture was incubated at 4 °C overnight. The antigen-containing pellet precipitate was collected by centrifugation at 20,000× *g* for 30 min at 4 °C and resuspended in one tenth of the original supernatant volume of phospate buffered saline (PBS) pH 7.2. The antigen was further dialysed against PBS to remove the ammonium suphate (3–4 buffer changes). The WAg preparation was aliquoted and stored at −70 °C until use. Uninfected Vero C1008 cells were prepared in the same way and used as control antigen.

#### 2.3.2. Prodution of Recombinant Ebola Virus Nucleoprotein

The codon optimized nucleotide sequence for EBOV nucleocapsid (NP) (GenBank accession number AF086833.2) was synthesized with a C-terminal glycine linker to a 6× Histidine tag (GenScript, Piscataway, NJ, USA) and subcloned into the NcoI and XhoI restriction sites of the pET-15b expression vector (Novagen, Merck, USA). The sequence verified plasmid (pET-15b ZEbov NP) was used to transform competent BL21 Star (DE3) *E. coli*. A starter culture was grown overnight at 37 °C, then diluted 10 fold and allowed to reach exponential-phase growth (OD_600_ of between 0.6–0.8) before protein expression was induced using 1 mM IPTG (Sigma Aldrich, Merck, USA) for 20 h at 37 °C with vigorous shaking. Cells were harvested by centrifugation, resuspended in sodium phosphate buffer (50 mM NaH_2_HPO_4_, 300 mM NaCl, pH 8.0) and lysed using a combination of Bugbuster and Lysonase (Novagen, Merck, USA) treatment, freeze-thaw cycles and sonication. The recombinant NP protein-containing soluble phase was collected by high speed centrifugation (15,000× *g*, 30 min, 4 °C) and loaded onto Profinity IMAC (Biorad, Hercules, CA, USA) cobalt-charged resin. The protein was left to bind overnight with gentle shaking at 4 °C, using a ratio of 1 mL resin to 16 mL soluble protein fraction. Contaminating proteins were removed by washing the resin twice with 20 packed-resin volumes of sodium phosphate buffer containing 12.5 mM imidazole (pH 8.0), using gentle centrifugation (1000× *g*, 2 min, 4 °C). The recombinant NP was then eluted overnight with gentle shaking at 4 °C in 2 packed-resin volumes of sodium phosphate buffer containing 250 mM imidazole (pH 8.0). Eluted protein was dialysed into 0.05 M carbonate/bicarbonate buffer, pH 9.6 (Sigma Aldrich, Merck, USA). To remove any residual contaminating proteins, the purified protein was passed through a SEC 650 size exclusion column (Biorad, Hercules, CA, USA) and the fractions containing the NP were collected. Collected fractions were pooled and concentrated using Amicon Ultra filters (Millipore, Merck, USA) with a 50 kD cut-off. The purified NP was quantified using the Bradford Concentration Assay kit (Pierce, ThermoFisher Scientific, Waltham, MA, USA) and aliquots were stored at −70 °C for later use. For the control antigen, the same process was followed using an expression vector (pET-15b) without an insert.

#### 2.3.3. Recombinant Ebola Virus Glycoprotein

Recombinant EBOV glycoprotein (GP) antigen expressed in human embryonic kidney 293 T cells was obtained from Integrated BioTherapeutics (Rockville, MD, USA).

#### 2.3.4. Indirect Enzyme-Linked Immunosorbent Assay Procedures

Optimal immunoreagents concentrations/working dilutions for I-ELISA were determined using standard checkerboard titration procedures [42]. Microtiter 96-wells plates (Maxisorb Immunoplates, Nunc, Roskilde, Denmark) for WAg, NP, and GP I-ELISAs were respectively coated with WAg and corresponding control antigen, NP and corresponding control antigen or with GP. For WAg and NP I-ELISAs virus and control antigens were added to rows of the top half of the plate (rows A–D:1–12) and the bottom half of the plate (rows E–H: 1–12), respectively. For GP I-ELISA all rows (rows A–H: 1–12) were coated with the commercial GP for which control antigen was not available. The NP and corresponding control antigen were diluted 1:2000 (stock concentration 0.4 mg/mL) in carbonate–bicarbonate buffer, pH 9.6. All the other antigens were diluted in phosphate PBS without Mg and Ca, pH 7.4; WAg and uninfected Vero C1008 cell culture antigen was diluted 1:300 and the GP was diluted 1:6000 (stock concentration 3.83 mg/mL).

Each 96-well plate had four replicates of positive serum control (C++), two replicates of negative serum control (C−) and two replicates of conjugate control (Cc). For WAg and NP I-ELISA C++ was added to wells A 1–2 and B 1–2 coated with virus antigen and to corresponding wells E 1–2 and F 1–2 coated with a control antigen. Accordingly, C− was added to C 1–2 and G 1–2, and Cc (diluent buffer) was added to D 1–2 and H 1–2. The remaining wells (A–D 3–12 and E–H 3–12) were used for testing 20 test sera in duplicate with test serum no. 1 added to wells A3 and B3 and corresponding E3 and F3; adding of the remaining test sera followed the same layout principle. For GP I-ELISA, not having control antigen, internal controls were placed as follows: C++ in wells A 1–2 and B 1–2; C− in wells C 1–2; Cc in wells D 1–2. The remaining wells (A–D 3–12 and E–H 1–12) were used for testing 44 test sera in duplicate with test serum no. 1 added to wells A3 and B3 and accordingly test serum no. 44 added to wells G12 and H12.

All reagents were added to the immunoplates at a volume of 100 µL/well unless otherwise stated. Passive adsorption onto ELISA plates was performed at 4 °C overnight and all subsequent incubations (except for substrate addition) were performed at 37 °C in a humidified chamber for 1 h. Following coating, plates were washed 3 times with 300 µL of PBS containing 0.1% Tween 20; the same washing procedure followed each subsequent stage of I-ELISAs. Plates were blocked with 200 µL 10% non-fat milk powder in PBS. After incubation, plates were washed, and control and test sera diluted 1:400 in 2% non-fat milk powder in PBS (diluent buffer) were added. Each test serum, negative control serum and conjugate control was tested in duplicate, and positive control was tested in quadruplicate. Following incubation with the sera, the plates were washed and goat anti-human IgG HRPO conjugate (Invitrogen, ThermoFisher Scientific, USA), diluted 1:10,000 in diluent buffer, was added to the wells. After incubation, plates were washed and 2,2’-azinodiethylbenzothiazoline sulfonic acid (ABTS) peroxidase (Seracare Lifesciences, Milford, MA, USA) substrate added to wells. Plates were incubated in the dark at room temperature (±22 °C) for 30 min.

Reactions were stopped by the addition of 1% sodium dodecyl sulphate (SDS) and the optical densities (OD) readings were measured at 405 nm. OD readings were converted into a percentage of the positive internal control serum (PP) using the equation as previously described [41]. Briefly, a specific activity of each serum (net OD) was calculated by subtracting the non-specific background OD in the wells with control antigen from the OD in wells with virus antigen (for NP and WAg I-ELISA only). The mean net OD readings were converted to PP using the formula: PP = (mean net of test serum/mean net OD of positive control) × 100 [41].

### 2.4. Selection of Cut-off Values and Statistical Analysis

Cut-off values were determined as mean +3 standard deviations (SD) of PP values recorded in SA and SL IgG EBOV negative serum panels and also selected at 95% accuracy level by the misclassification cost term (MCT) option of the two-graph receiver operating characteristics (TG-ROC) analysis available from Microsoft Excel 2010 (Redmond, WA, USA) [43,44] using SL reference IgG EBOV negative and positive serum panels. Coefficient of variation (CV) values were calculated to measure relative variability using the formula CV = (SD of replicates/mean of replicates) × 100.

Optimisation of cut-off values by MCT TG-ROC was based on the following equation: MCT = (1-p) (1-Sp) + rp (1-Se), where p (prevalence) = 0.5 and r (cost of false-positive and false-negative results) = 1.0. I-ELISAs diagnostic sensitivity (DSe) and specificity (DSp) were calculated as follows [45]: DSe = [TP/(TP + FN)] × 100; DSp = [TN/(TN + FP)] × 100; where TP = true positive sera (true EBOD patients), FN = false-negative sera (confirmed infected EBOD patients that tested negative), TN = true negative sera (individuals that truly are negative for EBOD), FP = false-positive (individuals negative for EBOD that test positive). Using Stata 13 (StataCorp, College Station, TX, USA), we obtained DSe and DSp statistics with their 95% confidence intervals for each of the diagnostic tests.

To compare the diagnostic performance of the assays and agreements between results of matched test samples, data were analyzed in Stata 13 using McNemar’s test and Cohen’s kappa statistic (κ) [46].

### 2.5. Serum Inactivation

To evaluate the effect of serum inactivation on the levels of the detectable anti-EBOV IgG by WAg, NP and GP I-ELISAs, laboratory protocols previously shown to completely inactivate EBOV virus in human serum were used [47,48]. Briefly, the C++ was first diluted 1:10 in PBS containing either 0.5% Triton X-100 or 0.5% Tween-20 (Sigma-Aldrich, Taufkichen, Germany) and heated at 60 °C for 15 min. Then, two-fold log_10_ dilutions (from 1:100 to 1:200,480; log_10_10^2^−log_10_10^5.3^) of untreated and treated serum was tested by each of the I-ELISAs. Each dilution was tested in duplicate on 3 separate runs. A serum titer was considered the highest sample dilution at which its PP value was ≥ the I-ELISA cut-off for at least 50% of six replicates.

## 3. Results

### 3.1. IQC and Repeatability

Both within and between the runs, the C++ OD readings for WAg, NP and GP I-ELISAs were within the IQC lower (LCL) and upper (UCL) control limits. Within the runs, the average CV ranged between 3.54 ± 1.99 (NP I-ELISA) and 4.76 ± 2.16 (GP I-ELISA). Between the runs, the average CV ranged between 3.11 ± 1.05 (NP I-ELISA) and 4.8 ± 1.56 (GP I-ELISA) (Table 1). Both within and between the runs, the C− and Cc OD readings were within the IQC LCL and UCL. For WAg, NP, and GP I-ELISA, the IQC C− OD control limits ranged from −0.08 to 0.06, −0.08 to 0.09, 0.1 to 0.17, and the IQC Cc− OD control limits ranged from −0.05 to 0.06, −0.05 to 0.04, and 0.03 to 0.11, respectively.

### 3.2. Distribution of I-ELISA PP Values in EBOV IgG Negative Sera and the Selection of Cut-off Values

**Figure 1:**
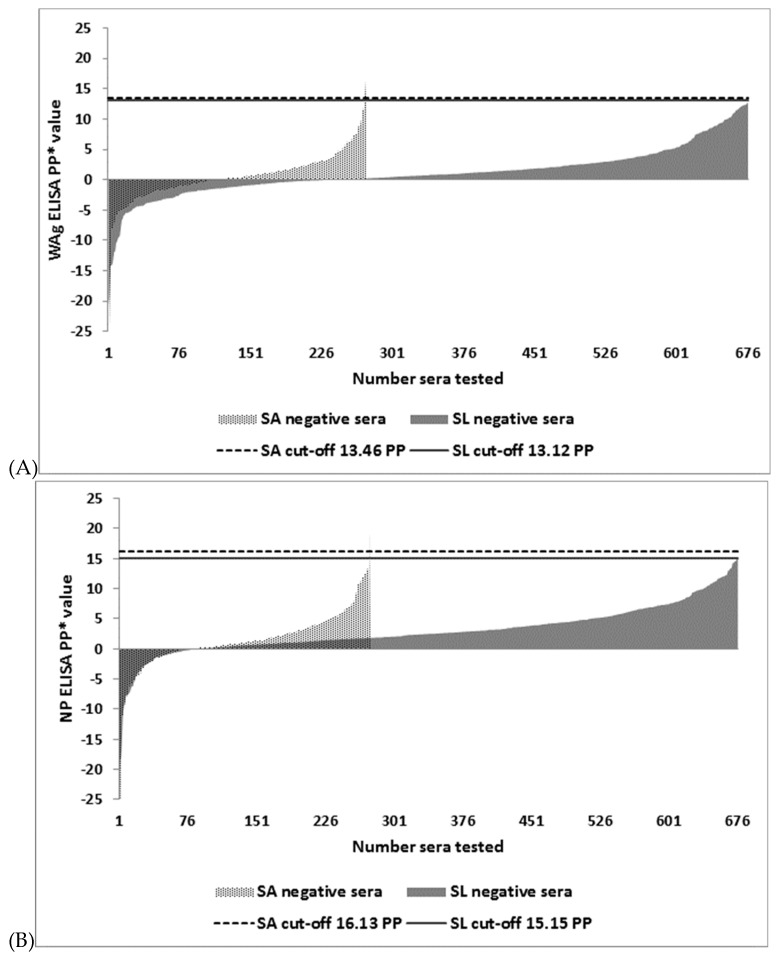

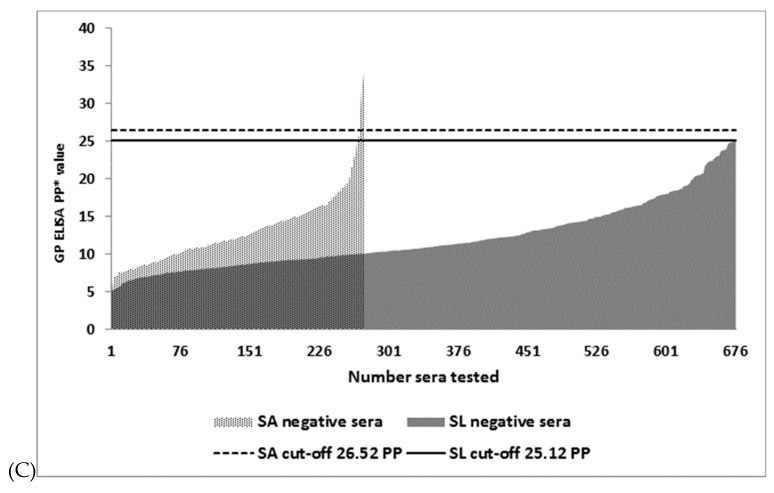
Distribution of percentage positivity (PP) values in South African (SA) and Sierra Leonean (SL) Ebola virus (EBOV) immunoglobulin G (IgG) negative human sera and selection of cut-off values for (**A**) the whole antigen (WAg), (**B**) nucleocapsid protein (NP) and (**C**) glycoprotein (GP) based ELISAs. Cut-off value for each assay was calculated as mean plus three standard deviations of ELISA PP (percentage positivity of internal positive control serum) values recorded by each test in 273 SA and 676 SL EVD negative sera, respectively.

**Figure 2:**
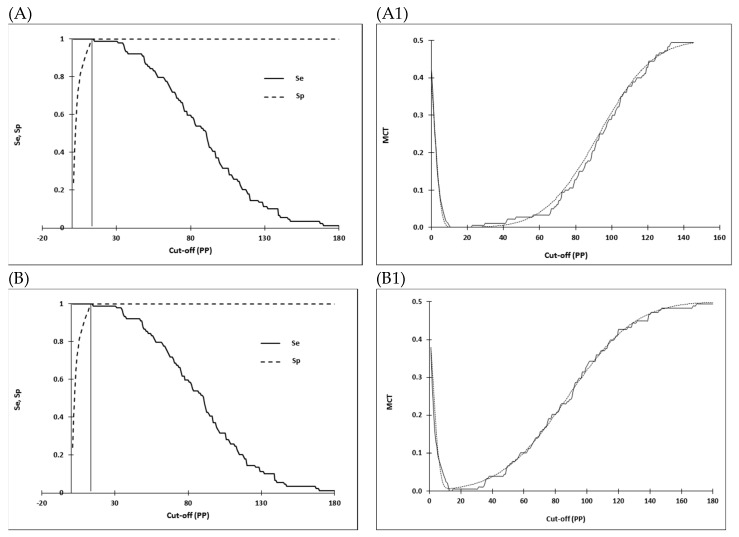

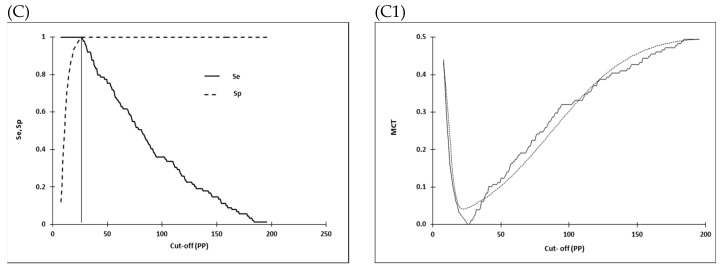
Optimization of cut-offs for Wag (**A**), nucleocapsid (**B**) and glycoprotein (**C**) ELISAs using the two-graph receiver operating characteristic analysis (TG-ROC). The insertion point of the sensitivity (Se, smooth line) and specificity (Sp, dashed line) graphs represents a cut-off PP value (13.53, 16.44 and 26.28, respectively) at which the highest and equivalent test parameters (Se = Sp) are achieved at 95% accuracy level. Using the misclassification cost term (MCT) option of the TG-ROC, at these cut-of values, the overall misclassification costs for WAg (**A1**), NP (**B1**), and GP ELISA (**C1**) become minimal (0.0003, 0.0069, 0.0001, respectively) under assumption of 50% disease prevalence and equal costs of false-positive and false-negative results. The two MCT curves represent values based on non-parametric (dashed line) or parametric (smooth line) estimates of Se and Sp derived from data sets analyzed. Optimization of cut-off values was based on the non-parametric program option due to departure from a normal distribution of data analyzed. Cut-off values are expressed as percentage positivity (PP) of an internal positive serum control.

The distribution of I-ELISA PP values and determination of cut-offs as mean plus three standard deviations of PP values recorded by each test in SA and SL EBOV IgG negative serum panels are shown in Figure 1. Selection and optimization of cut-offs by the MCT TG-ROC in SL serum panels is shown in Figure 2.

MCT TR-ROC was based on the non-parametric program option due to departure from a normal distribution of data sets analyzed. PP threshold values for each of the assays were similar irrespective of serum panels analyzed and the methods used for the determination of cut-offs: 13.46, 13.12 (Figure 1A) and 13.35 (Figure 2A) for WAg I-ELISA; 16.13, 15.15 (Figure 1B) and 16.44 (Figure 2B) for NP I-ELISA; 26.52, 25.12 (Figure 1C) and 26.28 (Figure 2C) for GP I-ELISA, respectively. The higher cut-off values for GP I-ELISA were likely due to higher ELISA noise resulting from not including a control antigen in this assay.

### 3.3. Comparison of D-Sp and D-Se

Irrespective of data set analyzed and cut-offs used, all the three assays evaluated in this study had high estimates of D-Sp, ranging from 97.4% to 99.6% in SA, and from 99.7% to 100% in SL negative serum panels. However, D-Sp for GP I-ELISA was generally lower in the SA negative serum panel (Table 2).

Individual results yielded by WAg, NP and GP I-ELISAs in sera from 423 RT-PCR confirmed EBOD cases at different times post disease onset and using different cut-off values are given in Table 3. Irrespective of the cut-offs used, the results were similar for the same assay, but the NP I-ELISA was more sensitive in detecting IgG antibody during the first two weeks post disease onset compared to WAg and GP I-ELISAs, the latter being the least sensitive. For example, when using the TG-ROC derived threshold, of 335 EBOD patients bled during the two weeks post onset, 92 (27.5%), 114 (34%), and 75 (22.4%) were positive by WAg, NP, and GP I-ELISA, respectively. Mc Nemar test indicate a disagreement of diagnostic capacity (combined DSe and DSp) between NP and WAg (*p* = 0.0196) or GP I-ELISA (*p* = 0.0002) respectively using all Sierra Leone data (*n* = 1099). Agreement was found between WAg and GP I-ELISA (*p* = 0.0707; 98.18% κ = 0.9300 ± 0.0348). Most of the discrepant (non-matching) results were recorded during the first two weeks post disease onset. After two weeks post onset, detection of IgG antibody and agreement between assays significantly improved. Of 88 sera tested 15–31 days post onset, all tested positive by WAg and NP I-ELISs irrespective of the cut-off used, and depending on the cut-off, 87 or 86 were positive by GP I-ELISA (Table 3). Mc Nemar test indicate agreement between NP, Wag and GP I-ELISAs ranging from 99.35% (κ = 0.9684 ± 0.0362) to 100% using data from non-EBOD and diseased patients bled on day 15 or later post-onset (*n* = 764), except for GP and Wag or NP I-ELISAs with the cut-offs of respectively 26.28, 13.53, 16.44 (*p* < 0.001).

Estimates of D-Se in sera collected at different times post disease onset are given in Table 4. Between 8–36 days post onset, the D-Se ranged from 82.6% (GP I-ELISA) to 92.65 (NP I-ELISA). Between 15–36 days post onset, the D-Se was 100% for both WAg and NP I-ELISA irrespective of the cut-off used, and ranged from 97.7% to 98.9% for the GP I-ELISA.

**Figure 3:**
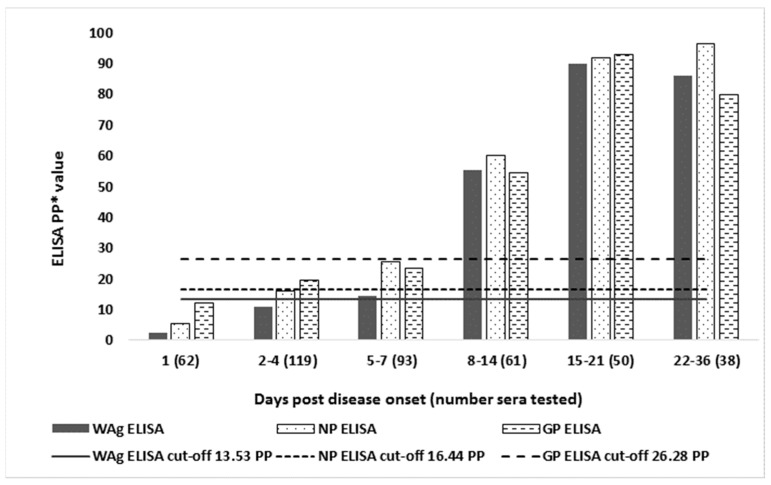
Mean IgG responses in 423 Ebola disease patients measured by a whole antigen (WAg), nucleocapsid (NP), and glycoprotein (GP) indirect ELISAs at different time post disease onset.

Mean levels of IgG responses measured by WAg, NP, and GP I-ELISAs in 423 EBOD RT-PCR confirmed cases bled at different times after disease onset are shown in Figure 3. On average, the first seroconversions were detectable by NP on days 2–4, then on days 5–7 by NP and WAg, and from day 8 post onset by all I-ELISAs. The mean levels of IgG measured by all the I-ELISAs were not significantly different.

**Figure 4:**
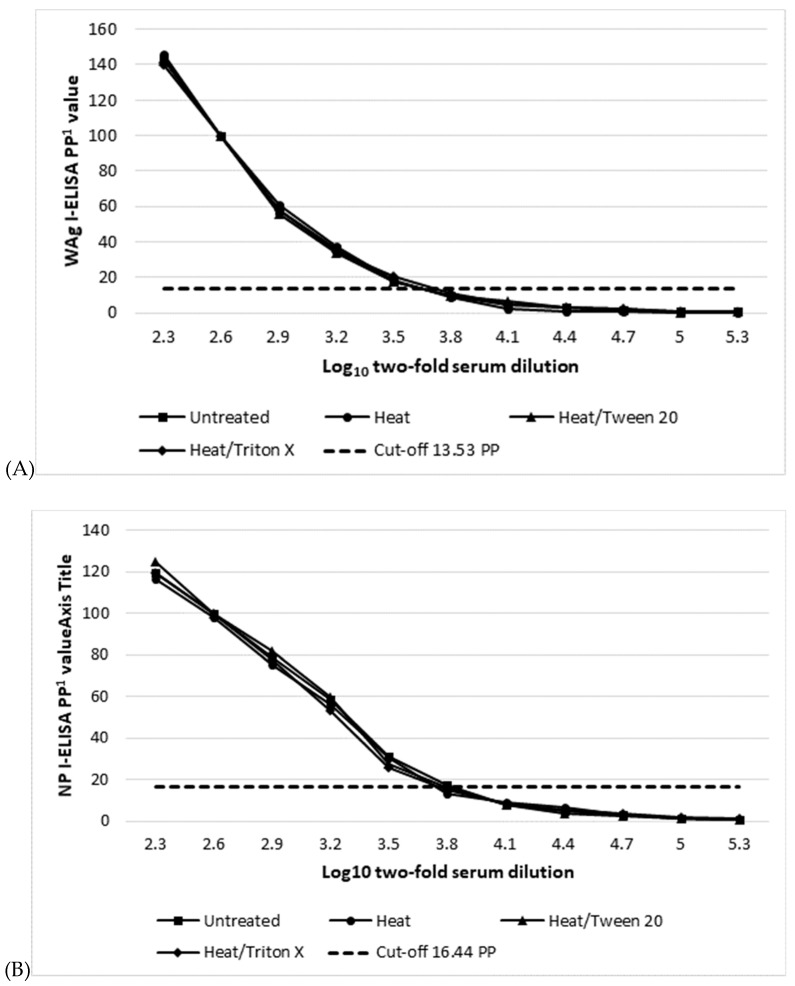

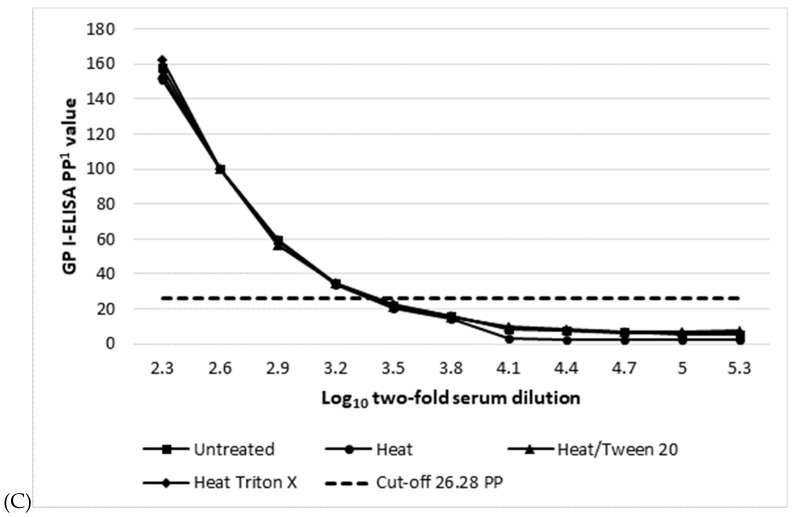
Dose-response kinetics of a positive EBOV IgG human serum before and after different inactivation procedures measured by a whole antigen (Wag), (**A**); nucleocapsid (NP), (**B**); and glycoprotein (GP), (**C**) indirect ELISA. ^1^ Percent positivity of internal positive control serum.

The different inactivation protocols used did not have an adverse effect on the kinetics and the detectable levels of the anti-EBOV IgG in C++ by either I-ELISA evaluated in this study. The titers (Table 5) as well as the kinetics of dose-response curves (Figure 4) were similar before and after inactivation in each assay.

## 4. Discussion

The diagnostic decision limit or cut-off represents a serological assay test value used to dichotomize negative and positive results, and by inference, to define the infection status of an individual against a specific pathogen of disease. The relevance of data used for the determination of cut-off consequently impacts on estimates of D-Se and D-Sp and other measures of test performance [49]. Important consideration in determining a serological assay cut-off is to select sera from unequivocally infected individuals and sera from individuals who have never been infected with the agent in question. Also, in order to account for the distribution of covariate factors (genetic, nutritional, geographical, and stage of infection) that may influence the estimates of D-Se and D-Sp, the target population should preferably be sampled using simple random, systematic or stratified sampling methods [45]. These ideal conditions could not be applied during this study. An assay validation data should preferably be derived not only from testing samples from reference individuals of known history and infection status but also from the country or region in which the test is to be used. Traditionally, gold standards for selection of truly infected and uninfected subjects include isolation of the agent or pathognomonic histopathological criteria. Because a true gold standard is difficult to accomplish, relative standards of comparison are often necessary, and include results from other serological assays [50]. In order to ensure EBOV true infection status of individuals whose sera were used in our study, RT-PCR negative sera that tested negative for anti-EBOV IgG by all IgG ELISAs using cut-offs derived from SA IgG EBOV negative reference serum panel were regarded as SL reference negative serum panel. Serum specimens from the SL EBOV RT-PCR positive patients with known dates of disease onset were regarded as SL reference positive serum panel.

Various statistical analyses used in our study for the selection of the cut-off values provided similar results. A cut-off value determined as two or three SD above the mean in uninfected individuals is frequently used for the interpretation of serodiagnostic tests. However, this assumes a normal distribution of the test values in population targeted by an assay, and provides only an estimate of D-Sp [49]. Deviations from normality are often recorded in serological data and should be addressed in the selection of threshold values [51]. Therefore, we also used the TG-ROC analysis for the selection and optimization of cut-off values to account for parametric versus nonparametric distribution of test values. All I-ELISAs evaluated in our study had high estimates of D-Sp, but the D-Se was dependent on the time when the serum was taken post disease onset. High detection rate of anti-EBOV IgG in RT-PCR EBOV confirmed cases was only recorded after two weeks post disease onset. Results of previous study in a small number of the 1995 EBOD patients in Kikwit, Democratic Republic of Congo, suggested that many patients do not have antibody early in the course of their illness, and that many may die without developing detectable antibodies to EBOV. Therefore, measurement of IgG antibody is of rather limited use in the diagnosis of acute EBOD cases [52].

I-ELISA, represents one of the simplest ELISA formats, but can be difficult to validate because of signal amplification of both specific and non-specific components [42]. For these reasons, to determine the specific binding of antibody, sera should be tested with both a specific viral antigen and its corresponding control or comparison antigen to account for possible non-specific background activity. The GP I-ELISA evaluated in our study was based on a commercially available recombinant GP for which a negative control could not be obtained. Compared to WAg and NP I-ELISAs, which included control antigens, the higher GP I-ELISA test values in EBOV negative serum panels and consequently the higher PP cut-off values derived for this assay are likely due to not having a control antigen. Due to inherent differences amongst assay systems, binding-antibody levels should be expressed in relative rather than absolute terms. One of the advantages of using PP values as a measure of antibody activity in the I-ELISA is that this method of OD readings conversion does not assume a uniform background activity, and therefore it is also more suitable for inter-laboratory standardization [53].

The first ELISAs for the detection of antibodies to EBOV were based on whole antigen prepared from infected cell lysate [40], and variations of this assay are still widely used in diagnostic and research laboratories [3]. While whole filovirus antigens can be relatively easily produced in large volumes, their preparation pose health risks, restricting their production to biosafety level 4 (BSL-4) facilities. These facilities are not only very expensive to construct and operate, but also are not easily available or accessible for countries where fatal filoviruses are endemic. In addition, the binding of antibodies to cellular contaminants present in EBOV-infected cell lysates may lead to cross-reactivity, resulting in reduced specificity [54]. High quality filovirus recombinant protein antigens can be safely prepared without the need for high BSL-4 biocontainment facilities and outside EBOV endemic areas. Their use in ELISA has potential to reduce the risk of false positive results and allows for better standardization [55,56]. Testing clinical specimens potentially containing a BSL-4 viral agent presents a serious biohazard. While a number of inactivation methods were shown to completely inactivate EBOV [47,48], they also markedly alter the protein components in human blood, e.g., enzymes and coagulation factors [47]. Results of viral inactivation protocols evaluated in our study indicate that they do not alter detectable levels of anti EBOV-IgG, thus together with recombinant antigen based ELISAs, provide a safe and reliable testing platform.

It has previously been reported that the use of single filoviral proteins as antigens is disadvantageous for filovirus serology [28,29,55]. Sera from patients infected with EBOV contain antibodies to several viral proteins and therefore might display reduced activity or later seroconversion in ELISAs based on a single recombinant antigen [55]. Results of our study indicate that anti-EBOV NP IgG is detectable earlier then anti-EBOV GP IgG antibody during the first week post disease onset. This is likely due to higher abundance of this highly immunogenic protein in infected cells [24].

Long-lasting persistence of IgG antibody in humans after infection with EBOV [40,57,58], renders IgG detection ELISA a suitable tool for epidemiological investigations.

Evaluation of the efficacy of filovirus vaccines and therapeutics require monitoring of immune responses that correlate with protection and survival using reliable and reproducible serological methods. Anti-EBOV GP IgG ELISA was recently shown to reproducibly quantify levels of anti-EBOV IgG antibodies in sera from EBOD survivors and immunized individuals [59], thus offering an important laboratory tool for assessing immunogenicity of candidate EBOV vaccines. The NP IgG I-ELISA evaluated in this study has a potential to be used for testing immunity and evaluating protection against natural EBOV exposure in individuals immunized with EBOV GP-based vaccines or receiving anti-EBOV GP passive immunization.

## 5. Conclusions

As highly accurate, robust and safe tests, the ELISAs based on recombinant EBOV antigens have a potential to replace traditional serological diagnostic methods which pose health risks thus necessitating their use only in high biocontainment facilities. Practically simple viral inactivation protocols evaluated in our study do not alter measurable levels of anti EBOV-IgG, thus together with recombinant antigen based ELISAs provide a safe testing application. Long-lasting IgG antibody makes their detection useful for epidemiological investigations.

## Figures and Tables

**Table 1:** Internal control quality data and repeatability estimates for a whole (WAg), nucleocapsid (NP), and glycoprotein (GP) indirect ELISAs for the detection of anti-IgG EBOV antibody in humans.

C++ ^1^	OD ^2^ IQC ^3^		Mean OD ± SD ^4^		Mean CV ^5^ ± SD (%)	
	LCL ^6^	UCL ^7^	Intra-Plate Variation	Inter-Plate Variation	Intra-Plate Variation	Inter-Plate Variation
WAg	0.81	1.29	1.00 ± 0.09	1.00 ± 0.08	4.26 ± 2.67	4.41 ± 1.85
NP	1.14	1.79	1.45 ± 0.18	1.50 ± 0.10	3.54 ± 1.99	3.11 ± 1.05
GP	0.9	1.39	1.19 ± 0.09	1.2 ± 0.04	4.76 ± 2.16	4.8 ± 1.56

^1^ Positive internal control serum; ^2^ Optical density; ^3^ Internal quality control; ^4^ Standard deviation; ^5^ Coefficient of variations; ^6^ Lower control limit; ^7^ Upper control limit.

**Table 2:** Diagnostic specificity of whole antigen (WAg), nucleocapsid (NP), and glycoprotein (GP) indirect ELISAs for the detection of anti-IgG EBOV antibody in humans.

Test	Cut-off PP ^1^	SA Negative Serum Panel (*n* = 273)		SL Negative Serum Panel (*n* = 676)	
		FP ^2^/TN ^3^	DSp ^4^ (95% CI ^5^)	FP/TN	DSp (95% CI)
WAg I-ELISA	13.46 ^6^	2/271	99.3 (97.4–99.9)	0/676	100 (99.5–100)
WAg I-ELISA	13.12 ^7^	2/271	99.3 (97.4–99.9)	0/676	100 (99.5–100)
WAg I-ELISA	13.53 ^8^	2/271	99.3 (97.4–99.9)	0/676	100 (99.5–100)
NP I-ELISA	16.13 ^6^	1/272	99.6 (98–100)	0/676	100 (99.5–100)
NP I-ELISA	15.15 ^7^	1/272	99.6 (98–100)	2/674	99.7 (98.9–100)
NP I-ELISA	16.44 ^8^	1/272	99.6 (98–100)	0/676	100 (99.5–100)
GP I-ELISA	26.52 ^6^	5/268	98.2 (95.8–99.4)	0/676	100 (99.5–100)
GP I-ELISA	25.12 ^7^	7/268	97.4 (94.8–99)	2/674	99.7 (98.9–100)
GP I-ELISA	26.28 ^8^	5/268	98.2 (95.8–99.4)	0/676	100 (995–100)

^1^ Percentage of the positive internal control serum; ^2^ False positive; ^3^ True negative; ^4^ Diagnostic specificity; ^5^ Confidence intervals; ^6^ Cut-off value determined as mean + 3 standard deviations of PP values recorded in SA IgG EBOV negative serum panel; ^7^ Cut-off value determined as mean +3 standard deviations of PP values recorded in SL IgG EBOV negative serum panel; ^8^ Cut-off value at 95% accuracy level determined by the two-graph receiver operating characteristics analysis using data from SL IgG EBOV negative and positive serum panels.

**Table 3:** Results of a whole antigen (WAg), nucleocapsid (NP), and glycoprotein (GP) indirect ELISAs for the detection of IgG antibody in 423 Ebola disease cases bled at different times post disease onset.

Days Post Disease Onset (Number Individuals Tested)	WAg No.pos. ^1^ (% pos.)	NP No. pos.(% pos.)	GP No. pos.(% pos.)	Only WAg pos.	Wag and NP pos.	Wag and GP pos.	Only NP pos.	NP and GP pos.	Only GP pos.
1 (*n* = 62)	3 ^2^ (4.8)	4 (6.5)	1 (1.6)	0	3	0	0	1	0
	3 ^3^ (4.8)	5 (8.1)	2 (3.2)	0	3	0	1	1	1
	3 ^4^ (4.8)	4 (6.5)	1 (1.6)	0	3	0	0	1	0
2–4 (*n* = 119)	20 (16.8)	27 (22.7)	13 (11.0)	1	6	0	8	0	0
	20 (16.8)	28(23.5)	13 (11.0)	1	6	0	9	0	0
	20 (16.8)	27 (22.7)	13 (11.0)	1	6	0	8	0	0
5–7 (*n* = 93)	27 (29.0)	34 (36.6)	24 (25.8)	1	7	0	6	2	3
	27 (29.0)	34 (36.6)	26 28.0)	1	7	0	5	3	4
	27 (29.0)	34 (36.6)	24 (25.8)	1	7	0	6	2	3
8–14 (*n* = 61)	42 (68.9)	49 (80.3)	37 (60.7)	1	8	0	5	4	0
	43 (70.5)	50 (82.0)	39 (64.0)	1	9	0	3	6	0
	42 (68.9)	49 (80.3)	37 (60.7)	1	8	0	5	4	0
15–21 (*n* = 50)	50 (100)	50 (100)	49 (98.0)	0	0	0	0	0	0
	50 (100)	50 (100)	49 (98.0)	0	0	0	0	0	0
	50 (100)	50(100)	49 (98.0)	0	0	0	0	0	0
22–36 (*n* = 38)	38 (100)	38 (100)	37 (97.4)	0	0	0	0	0	0
	38 (100)	38(100)	38 (100)	0	0	0	0	0	0
	38 (100)	38 (100)	37 (97.4)	0	0	0	0	0	0

^1^ Positive result; ^2^ I-ELISA results using cut-off value determined as mean +3 standard deviations of ELISA readings recorded in South African IgG EBOV negative serum panel; ^3^ I-ELISA results using cut-off value determined as mean +3 standard deviations of ELISA readings recorded in Sierra Leonean IgG EBOV negative serum panel; ^4^ I-ELISA results using cut-off value determined by the two-graph receiver operating characteristics analysis of ELISA readings in Sierra Leonean IgG EBOV negative and positive serum panels.

**Table 4:** Diagnostic sensitivity of a whole antigen (WAg), nucleocapsid (NP), and glycoprotein (GP) I-ELISAs for the detection of anti-IgG EBOV antibody in humans.

Test	Cut-off PP ^1^	SL EBOV RT-PCR Positive Cases Days 8–36 Post Onset (*n* = 149)		SL EBOV RT-PCR Positive Cases Days 15–36 Post Onset (*n* = 88)	
		FN ^2^/TP ^3^	DSe ^4^ (95% CI ^5^)	FN/TP	DSe (95% CI)
WAg I-ELISA	13.46 ^6^	19/130	87.2 (80.8–92.1)	0/88	100 (95.9–100)
WAg I-ELISA	13.12 ^7^	18/131	87.9 (81.6–92.7)	0/88	100 (95.5–100)
WAg I-ELISA	13.53 ^8^	19/130	87.2 (80.8–92.1)	0/88	100 (95.5–100)
NP I-ELISA	16.13 ^6^	12/137	91.9 (86.4–95.8)	0/88	100 (95.5–100)
NP I-ELISA	15.15 ^7^	11/138	92.6 (87.2–96.3)	0/88	100 (95.9–100)
NP I-ELISA	16.44 ^8^	12/137	91.9 (86.4–95.8)	0/88	100 (95.5–100)
GP I-ELISA	26.52 ^6^	26/123	82.6 (75.5–88.3)	2/86	97.7 (92–99.7)
GP I-ELISA	25.12 ^7^	23/126	84.6 (77.7–90)	1/87	98.9 (93.8–100)
GP I-ELISA	26.28 ^8^	26/123	82.6 (75.5–88.3)	2/86	97.7 (92–99.7)

^1^ Percentage of the positive internal control serum; ^2^ False negative; ^3^ True positive; ^4^ Diagnostic sensitivity; ^5^ Confidence intervals; ^6^ Cut-off value determined as mean +3 standard deviations of PP values recorded in SA IgG EBOV negative serum panel; ^7^ Cut-off value determined as mean + 3 standard deviations of PP values recorded in SL IgG EBOV negative serum panel; ^8^ Cut-off value at 95% accuracy level determined the two-graph receiver operating characteristics analysis using data from SL IgG EBOV negative and positive serum panels.

**Table 5:** Comparison of whole antigen (WAg), nucleocapsid (NP) and glycoprotein (GP) indirect ELISAs mean titers and analytical slopes of non-treated versus inactivated IgG EBOV positive serum.

Assay (Cut-off PP Value) ^1^	Mean log_10_ Serum Titre ^2^/Dose Response Curve R Square ^3^			
	Untreated	60° 1 h	0.5%Tween 20 15 min 60°	0.5%Triton X-100 15 min 60°
WAg I-ELISA (13.53)	3.5/0.945	3.5/0.950	3.5/0.942	3.5/0.952
NP I-ELISA (16.44 PP)	3.8/0.959	3.5/0.961	3.5/0.956	3.5/0.957
GP I-ELISA (26.28)	3.2/0.932	3.2/0.942	3.2/0.924	3.2/0.928

^1^ TG-ROC derived cut-off used; ^2^ Log_10_ highest serum dilution at which at least 50% of six replicates still tested positive; ^3^ Coefficient of determination.
